# Comparison of Magnetic Resonance Imaging with Electrodiagnosis in the Evaluation of Clinical Suspicion of Lumbosacral Radiculopathy

**DOI:** 10.3390/diagnostics14121258

**Published:** 2024-06-14

**Authors:** Alberto Montaner-Cuello, Santos Caudevilla-Polo, Diego Rodríguez-Mena, Gianluca Ciuffreda, Pilar Pardos-Aguilella, Isabel Albarova-Corral, Jorge Pérez-Rey, Elena Bueno-Gracia

**Affiliations:** 1 Department of Physiatry and Nursing, Faculty of Health Sciences, University of Zaragoza, Calle Domingo Miral S/N, 50009 Zaragoza, Spain; scp@unizar.es (S.C.-P.); gciuffreda@unizar.es (G.C.); ppardos@unizar.es (P.P.-A.); ialbarova@unizar.es (I.A.-C.); ebueno@unizar.es (E.B.-G.); 2 PhysiUZerapy: Health Sciences Research Group, University of Zaragoza, Calle Domingo Miral S/N, 50009 Zaragoza, Spain; drodriguezm@salud.aragon.es (D.R.-M.); jorge.perez@unizar.es (J.P.-R.); 3 Department of Neurophysiology, University Clinical Hospital “Lozano Blesa”, 50009 Zaragoza, Spain

**Keywords:** lumbosacral radiculopathy, magnetic resonance imaging (MRI), electrodiagnostic studies (EDX), diagnostic tests

## Abstract

(1) Background: The diagnosis of lumbosacral radiculopathy involves anamnesis, an assessment of sensitivity and strength, diagnostic imaging—usually magnetic resonance imaging (MRI)—and electrodiagnostic testing (EDX), typically electromyography (EMG), and electroneurography (ENG). MRI evaluates the structures supporting the spinal cord, while EDX evaluates root functionality. The present study aimed to analyze the concordance of MRI and EDX findings in patients with clinically suspected radiculopathy. Additionally, we investigated the comparison between these two reference tests and various clinical variables and questionnaires. (2) Methods: We designed a prospective epidemiological study of consecutive cases with an observational, descriptive, cross-sectional, and double-blind nature following the STROBE guidelines, encompassing 142 patients with clinical suspicion of lumbosacral radiculopathy. (3) Results: Of the sample, 58.5% tested positive for radiculopathy using EDX as the reference test, while 45.8% tested positive using MRI. The comparison between MRI and EDX in the diagnosis of radiculopathy in patients with clinical suspicion was not significant; the overall agreement was 40.8%. Only the years with symptoms were comparatively significant between the positive and negative radiculopathy groups as determined by EDX. (4) Conclusion: The comparison between lumbar radiculopathy diagnoses in patients with clinically suspected pathology using MRI and EDX as diagnostic modalities did not yield statistically significant findings. MRI and EDX are complementary tests assessing different aspects in patients with suspected radiculopathy; degeneration of the structures supporting the spinal cord does not necessarily imply root dysfunction.

## 1. Introduction

Radiculopathy is a dysfunction of a spinal nerve root that can cause pain, weakness, sensory disturbances, and/or decreased myotatic reflexes in a specific anatomical territory corresponding to the level of the affected root [1,2,3,4,5]. When the involved roots correspond to the lumbar and/or sacral spinal nerves, the term ‘lumbosacral radiculopathy’ or ‘lumbosacral radicular syndrome’ is commonly used [6,7]. The etiology by which a lumbosacral root can become affected is often related to mechanical and/or chemical phenomena [2,8,9,10]. Mechanical injury to the nerve root can occur due to compression, traction, or frictional forces. Chemical irritation may occur in response to ischemia of the nerve root, vascular stasis, or exposure of the root to inflammatory components released during tissue injury [8,11,12]. The prevalence of this pathology ranges from 1 to 5% of the general population [7,13,14].

The diagnosis of radiculopathy requires correlation of the results from different components of the evaluation process [11,13]. Imaging diagnosis, typically magnetic resonance imaging (MRI), and electrodiagnostic tests (EDX) are the two most commonly used reference tests to confirm nerve root damage [10,11,13,15].

The current diagnostic paradigm is overly mechanistic, focusing on structural damage that confirms disc herniation or degeneration of the structures housing the spinal cord and roots—the two major causes of radiculopathy [1,5,9,16,17,18]. This perspective results in MRI carrying significant weight in the diagnostic process. However, radiculopathy is a condition that affects the physiology of the nerve root, which may not necessarily be solely of compressive etiology [8,19]. Therefore, EDX—which allows for the physiological assessment of the root and detects alterations in function—is another essential test [20,21,22,23,24].

EDX exhibits high diagnostic accuracy when neurological symptoms have been present for at least three weeks, with a very low percentage of false positives. However, it can yield false negatives under circumstances of minimal demyelinating lesions, the involvement of very small fibers, or selective dorsal root involvement [21]. Although the accuracy of MRI in detecting structural abnormalities is well-established, the relationship between these detected anatomical anomalies and the signs and symptoms of patients remains controversial [4,10,25,26]. In one-third of patients diagnosed with radicular pain syndrome, no radicular compression is observed on MRI [6,19].

Some studies published to date coincide in reporting a low level of agreement between both reference tests [23,27,28,29], while some of these studies analyze EDX only in patients considered positive on MRI [23], include both cervical and lumbar radiculopathies [27,28], or consider back pain alone as sufficient clinical suspicion [30]. In patients with a clear clinical presentation and imaging evidence of mechanical compression, a good level of agreement is presumable. However, in undiagnosed patients with clinical suspicion, what level of correlation could we expect between both diagnostic tests?

The aim of this study was to analyze the degree of agreement between the two most commonly used reference tests in the diagnosis of lumbosacral radiculopathy in patients with clinical suspicion of the pathology and to compare them with other clinical variables and scales.

## 2. Materials and Methods

A prospective epidemiological study of consecutive observational, descriptive, cross-sectional, and double-blind cases (neither the patient nor the principal investigator knew the results of the other diagnostic test) was designed. The design and execution of this study followed the guidelines of the STROBE [31,32] (Strengthening the Reporting of Observational Studies in Epidemiology) guide for the reporting of observational studies.

The study population focused on patients referred to the Clinical Neurophysiology Service of the University Clinical Hospital “Lozano Blesa” in Zaragoza due to suspicion of lumbosacral radiculopathy to undergo EDX study—electromyography (EMG) and electroneurography (ENG)—for diagnostic confirmation.

The sample size calculation considered an approximate prevalence of 50% of the pathology in the study population, a 95% confidence interval, and a margin of error of 10% for similar values of sensitivity and specificity between 75% and 80%, resulting in a sample size of 142 patients [33]. 

A consecutive sampling method was applied to select patients referred for an EDX (EMG–ENG) study due to clinical suspicion of lumbosacral radiculopathy over a period of two years. Patients were informed about the study and provided consent for participation before inclusion. Inclusion criteria were as follows: patients aged between 18 and 80 years [34] presenting symptoms consistent with lumbosacral radiculopathy for more than 3 weeks at the time of the study—intermittent or constant pain in the lumbar area or radiating to a distal extremity, to the gluteal fold, or distribution of pain according to a dermatomal pattern or weakness according to a myotomal pattern of some lumbosacral nerve root—[35,36] and having sufficient understanding and communicative capacity to communicate their symptoms, as well as their characteristics [37]. The main exclusion criteria for participation were as follows: any diagnosis of type I diabetes mellitus, unregulated thyroid dysfunctions, rheumatoid arthritis, serious heart and/or lung diseases, herpes zoster infection, multiple sclerosis, hereditary neuropathy, known pregnancy, and/or serious systemic or autoimmune diseases [35,37,38,39,40], as well as having undergone lumbar spine surgery or suffered fractures in the lumbar spine in the last year [37].

Among the patients who met the selection criteria, data collection from their medical history was conducted. Demographic variables and information related to the pathology were recorded during the initial assessment. Additionally, patients completed the Oswestry Disability Index (ODI) scale [41], the Neuropathic Pain Diagnostic Questionnaire (DN4) [42], and the Visual Analog Scale (VAS) [43,44] to assess their current pain, with values recorded in writing. Subsequently, upon completion of the entire evaluation process and with the patient’s signed consent, the lumbar MRI report for each patient was obtained.

The EDX included EMG and ENG. EMG was performed using a concentric needle electrode. The ‘H’ reflex was also recorded, and ENG was utilized to exclude other diseases and confirm the diagnosis. The algorithm established by the American Association of Electrodiagnostic & Neuromuscular Medicine (AANEM) was followed to perform the EDX as outlined in the main manuals [21], which involves exploring the innervated muscles at the segmental level corresponding to the suspicion of radiculopathy. Muscle electrical activity was recorded at rest, during movement, and with submaximal effort. Pathological EMG findings included positive sharp waves, fibrillation potentials, complex repetitive discharges, high amplitudes, broad duration, increased polyphasic motor units, or reduced neuropathic recruitment [21,45]. Additional muscles with the same segmental innervation were studied to confirm the diagnosis if abnormalities were detected in one of the initially examined muscles. Additionally, this study was completed with ENG to determine if this abnormality was due to mononeuropathy. If none of the muscles exhibited abnormal findings, radiculopathy was ruled out [46].

The diagnosis of radiculopathy was considered positive when pathological findings were observed in at least 2 muscles sharing a common nerve root but originating from different peripheral nerves, and/or when the ‘H’ reflex was positive with abolished or attenuated response for S1 root [21,47,48].

Regarding the lumbar MRI reports, variables related to vertebral body degeneration, presence of osteophytes, spondylolisthesis, arthropathies, facet hypertrophy, foraminal stenosis, and different levels of disc degeneration (bulges, protrusions, extrusions) were recorded, with radiculopathy noted when the report explicitly referenced root involvement in any of these circumstances, following the main guidelines [49,50,51,52,53].

The statistical analysis of all variables was conducted using IBM^®^ SPSS^®^ Statistics 21. A confidence level of 95% was established for the analysis of the results and statistical inference. Descriptive statistics were presented as mean and standard deviation for continuous variables and frequencies for categorical variables. Comparisons of quantitative variables with MRI and EDX were conducted using the Mann–Whitney U test as a non-parametric test and the Student’s *t*-test with Levene’s test for parametric analysis. The association between EDX findings, and MRI findings was examined using the Chi-Squared test, with a *p*-value of <0.05 considered significant [54].

## 3. Results

**Figure 1:**
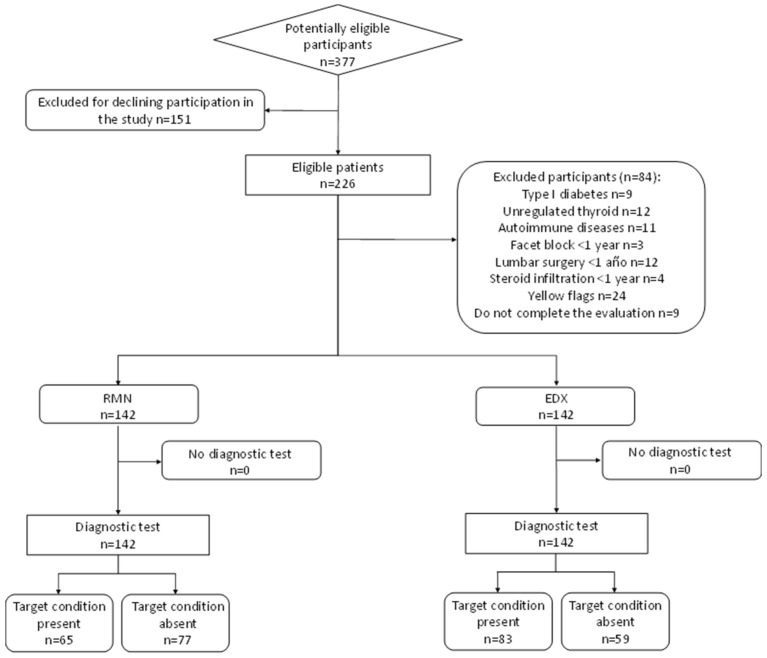
Recruitment Sample Flowchart.

An electrodiagnostic study and magnetic resonance imaging were conducted on 142 patients suspected of lumbar radiculopathy; Figure 1 presents the flow diagram. The sample comprised 60 (42.3%) males and 82 (57.7%) females, aged between 18 and 77 years, with a mean age of 54.82 years (SD 12.33). The mean body mass index (BMI) was 26.62 (SD 4.30), with 39.4% of the sample classified as overweight and 20.4% as pathologically obese. The mean duration of symptoms was 5.44 years (SD 6.10). Regarding the DN4 variable, the mean score was 3.44 points (SD 1.8), and considering a positive score from 4 points onwards [42], it was positive for 47.2% of the sample and negative for 52.8%. The mean score on the ODI scale was 34.33 points (SD 18.12). Following the classification of this scale [55], 28.9% of the sample had mild disability, 35.9% had moderate disability, 28.2% had severe disability, 6.3% had profound disability, and only 0.7% had very profound disability. Current pain, measured using the VAS scale, had a mean of 4.02 points (SD 2.47). 

**Figure 2:**
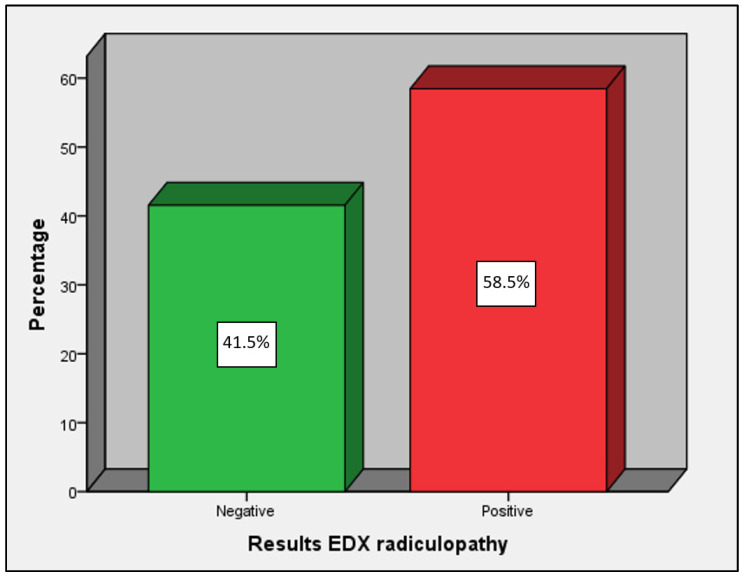
Results of EDX diagnosis of radiculopathy.

**Figure 3:**
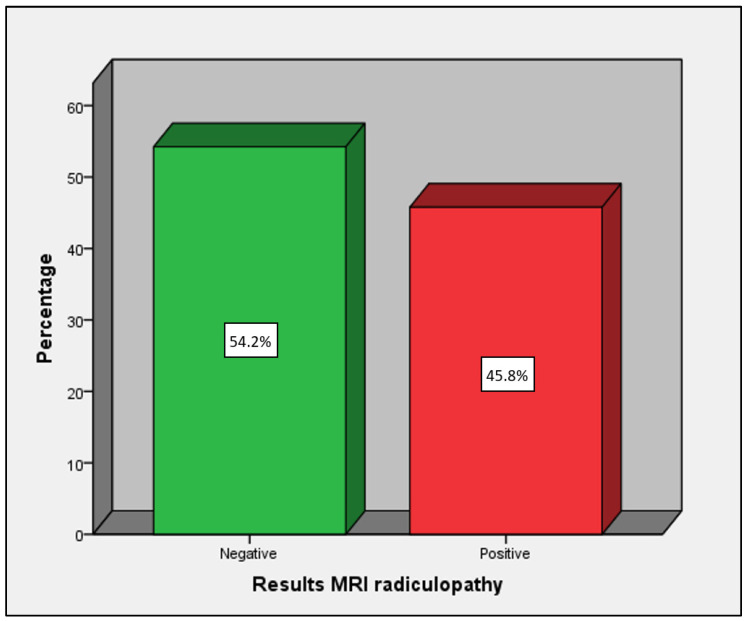
Results of MRI diagnosis of radiculopathy.

The results of the EDX were positive for radiculopathy in 58.5% and negative in 41.5% of cases (Figure 2). The L5 root was the most affected, accounting for 41.5%, followed by S1 with 26.1%. The MRI results showed that 45.8% tested positive for radiculopathy on MRI, while 54.2% tested negative (Figure 3). Furthermore, 79.6% of the patients had some type of disc herniation and 66.2% presented signs of lumbosacral arthropathy; the most damaged disc was L4, affecting 63.5% of cases, followed by L5 with 61.3%. 

Only the comparison of the variable ‘years with symptoms’ between the positive and negative radiculopathy groups via EDX was significant (*p* = 0.026) (Table 1); however, it was not significant using MRI. The remaining clinical variables analyzed (current pain VAS, ODI scale, and DN4) did not show comparative significance using either EDX (Table 1) or MRI (Table 2).

**Figure 4:**
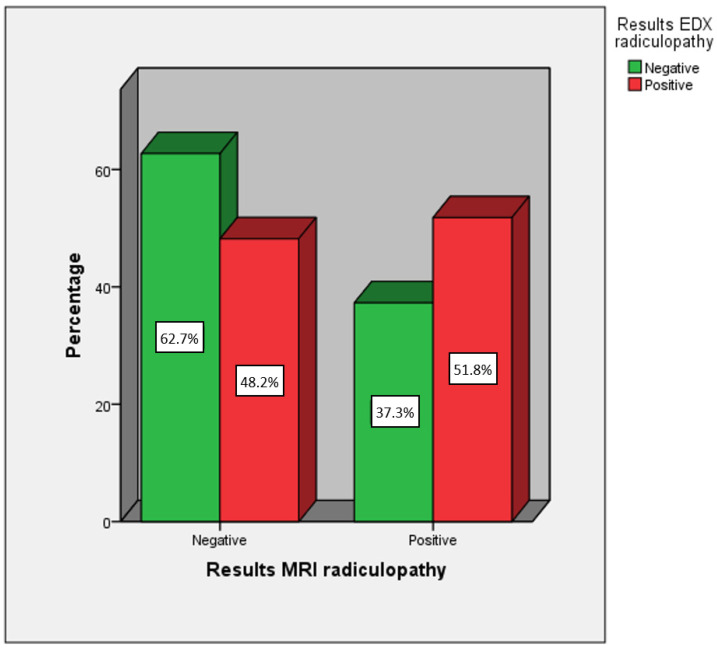
Comparison between positives and negatives for radiculopathy according to MRI and EDX.

The comparison between the variable MRI radiculopathy and EDX radiculopathy was not statistically significant (*p* = 0.087). Of those diagnosed as positive for radiculopathy using MRI, only 37.3% tested positive according to EDX; among those diagnosed as negative for radiculopathy using MRI, 62.7% tested negative according to EDX. The diagnostic agreement between both diagnostic tests, when seeking concordance in detecting radiculopathy on the same side and at the same lumbosacral segmental level, was 40.8% of cases (Figure 4 and Table 3).

## 4. Discussion

Our study first analyzed whether the comparison of different clinical variables between the positive and negative groups for radiculopathy using electromyography (EDX) or magnetic resonance imaging (MRI) as diagnostic tests was significant. Pain measured with the VAS at the time of examination resulted in the positive group showing lower levels compared with other similar studies, where significance was not observed, similar to our study using EDX as a reference [56,57]. The ODI scale also did not show statistical significance in the comparison using EDX; the recorded values were lower than in other studies [36,58]. Furthermore, the negative radiculopathy group had a higher mean than the negative group. Savage et al. [59] in their EDX study reported similar data, where the positive group scored 37.2 compared with 43.4 in the negative group. The DN4 questionnaire also did not show significant differences when comparing the positive and negative groups for radiculopathy in our study, similar to the findings of Savage et al. [59].

The only clinical variable that proved significant was ‘years with symptoms’ with the highest value observed in the positive group—6.64 (SD 7.21)—compared with 3.73 (SD 3.44) in the negative group. In many studies, the subject sample typically exhibits, on average, a shorter duration of symptoms, and we lack data for comparison. This finding would align with studies suggesting that a history of recurrent episodes of low back pain is a risk factor for developing radiculopathy [4,5,60,61].

The lack of significant results in any of the clinical variables—DN4, ODI scale, and current pain—when comparing positives and negatives for radiculopathy is consistent data in a sample with clinical suspicion of the pathology but without confirmation. This circumstance results in the inclusion of patients with other pathologies exhibiting concurrent symptoms, which may introduce confounding effects and complicate diagnosis [62,63,64].

Regarding the agreement in the diagnosis of lumbosacral radiculopathy between MRI and EDX, various studies have presented differing results. Reza et al. [27] compared the level of concordance between these two tests for lumbosacral radiculopathy, finding that 71% of MRIs showed findings consistent with radiculopathy, while EDX indicated 58%. Overall, the agreement level between the two tests was 59.6%. In the study by Yousif et al. [23], among subjects with suspected radiculopathy—excluding subjects from a control group not considered for this calculation—true negative and true positive concordance was 56.67%, which is very similar to our study and consistent with earlier studies [28]. In a recent study by Murtaza et al. [30], no significant relationship was found between both diagnostic tests, reinforcing the results obtained in our study.

In summary, it can be emphasized that both reference tests have their pros and cons, as MRI seeks structural damage while EDX detects alterations in the function of the neural system. As we can observe, the current mechanistic paradigm of seeking structural damage to confirm disc herniation or degeneration of structures housing the spinal cord and roots [1,5,9,16,17,18] through MRI does not align with the physiological study of the root using EDX. Currently, these are two diagnostic tests that assess different aspects of the pathology. Therefore, EDX, which allows for a physiological assessment of the nerve root and detects functional abnormalities in injuries lasting more than three weeks [21,24], will be a crucial test to confirm functional involvement of the nerve root when clinical suspicion and a positive MRI coexist [20,21,22,23,24].

## 5. Conclusions

The number of years with pain symptoms is a significantly related factor to the diagnosis of lumbosacral radiculopathy via EDX in patients with clinical suspicion of the pathology but not via MRI. Neither the ODI scale, DN4, nor current pain are significantly related factors to the diagnosis of lumbosacral radiculopathy in patients with clinical suspicion of the pathology using MRI or EDX as reference tests. 

The comparison between the diagnosis of lumbosacral radiculopathy in patients with clinical suspicion of the pathology using MRI and EDX as diagnostic tests did not yield statistically significant results. MRI and EDX are complementary tests assessing different aspects in patients with suspected radiculopathy; degeneration of the structures supporting the spinal cord does not necessarily imply root dysfunction.

## Figures and Tables

**Table 1:** Clinical Variables Comparison with EDX Radiculopathy.

	Radiculopathy (*n* = 83)		No Radiculopathy (*n* = 59)		
	Mean ± SD	CI 95%	Mean ± SD	CI 95%	*p* Value *
Years with symptoms	6.64 ± 7.21	5.06–8.22	3.76 ± 3.44	2.86–4.65	0.026
DN4	3.49 ± 1.84	3.09–3.90	3.36 ± 1.81	2.88–3.83	0.527
ODI scale	33.01 ± 17.87	29.11–36.91	36.19 ± 18.46	31.37–41.00	0.305
Current pain	3.76 ± 2.48	3.22–4.31	4.38 ± 2.42	3.75–5.01	0.144

* significant *p*-value < 0.05.

**Table 2:** Clinical Variables Comparison with MRI Radiculopathy.

	Radiculopathy (*n* = 83)		No Radiculopathy (*n* = 59)		
	Mean ± SD	CI 95%	Mean ± SD	CI 95%	*p* Value *
Years with symptoms	5.53 ± 6.58	3.90–7.16	5.37 ± 5.70	4.07–6.66	0.579
DN4	3.34 ± 1.72	2.91–3.76	3.52 ± 1.92	3.08–3.95	0.672
ODI scale	33.55 ± 17.20	29.29–37.81	34.99 ± 18.96	30.68–39.29	0.640
Current pain	3.60 ± 2.38	3.01–4.19	4.37 ± 2.50	3.81–4.94	0.630

* significant *p*-value < 0.05.

**Table 3:** Comparison of radiculopathy diagnosis between MRI and EDX.

	EDX Positive	EDX Negative	Total	*p* Value *
MRI positive	43 (51.8%)	22 (37.3%)	65 (100%)	0.087
MRI negative	40 (48.2%)	37 (62.7%)	77 (100%)	
Total	83 (100%)	59 (100%)	142 (100%)	

* Chi-square: significant *p*-value < 0.05.

## Data Availability

The data presented in this study are available upon request from the corresponding author. The data are not publicly available because permission was obtained only for their use in this study and not for public dissemination. These are pseudo-anonymized data that belong to the clinical records of each patient in the study.
